# BIM-Sim: Interactive Simulation of Broadband Imaging Using Mie Theory

**DOI:** 10.3389/fphy.2017.00005

**Published:** 2017-02-20

**Authors:** Sebastian Berisha, Thomas van Dijk, Rohit Bhargava, P. Scott Carney, David Mayerich

**Affiliations:** 1 Department of Electrical and Computer Engineering, University of Houston, Houston, TX, USA; 2 Department of Medical Physics, Máxima Medical Centre, Veldhoven, Netherlands; 3 Beckman Institute for Advanced Science and Technology, University of Illinois at Urbana-Champaign, Urbana, IL, USA

**Keywords:** mid-infrared, FTIR, QCL, imaging, scattering, Mie, GPU, Monte-Carlo

## Abstract

Understanding the structure of a scattered electromagnetic (EM) field is critical to improving the imaging process. Mechanisms such as diffraction, scattering, and interference affect an image, limiting the resolution, and potentially introducing artifacts. Simulation and visualization of scattered fields thus plays an important role in imaging science. However, EM fields are high-dimensional, making them time-consuming to simulate, and difficult to visualize. In this paper, we present a framework for interactively computing and visualizing EM fields scattered by micro and nano-particles. Our software uses graphics hardware for evaluating the field both inside and outside of these particles. We then use Monte-Carlo sampling to reconstruct and visualize the three-dimensional structure of the field, spectral profiles at individual points, the structure of the field at the surface of the particle, and the resulting image produced by an optical system.

## 1. INTRODUCTION

Particle interactions within an incident electromagnetic (EM) field are difficult to simulate and visualize because the result is inherently high-dimensional. EM radiation is generally represented using a complex vector field in three spatial dimensions. When the incident light consists of broadband radiation, each wavelength *λ* interacts with materials in a unique way. Studying the behavior of EM fields generates a time-dependent component, as the user changes properties of the particles or incident light in order to study these effects on the scattered field. Accounting for all of these factors requires a five-dimensional (5D) simulation in (*x, y, z, λ, t*), which is both computationally expensive and requires a prohibitive amount of memory.

In this paper, we propose software for interactive simulation of broadband Mie scattering in EM fields. We describe a mathematical model based on Mie theory that allows efficient evaluation of the scattered field using hardware and algorithms that support highly parallel computation. The high-dimensional nature of the simulation is constrained by user-specified visualization parameters. By taking advantage of GPU-based computation, we allow the user to interactively adjust these parameters to perform the simulation in real-time. This has several advantages over traditional simulation of the complete 5D forward model.

### 1.1. Applications

Mie theory plays an important role in describing the propagation of EM radiation by providing a rigorous solution to Maxwell’s Equations for a spherical scatterer. This theory has been used to characterize absorption in atmospheric simulations [1], and to approximate cellular structures in spectroscopic imaging of biological tissues [2]. There is also interest in using micro and nanospheres, such as quantum dots, to improve sensing in biomedicine [3]. Generalized Mie theory [4, 5] has been used in plasmonics applications to understand the near-filed response of nanoparticle aggregates to circularly polarized light [6, 7].

Mie theory is often used as a first-order approximation for scattering by general particles [8]. Of particular interest is the problem of inverse scattering in samples composed of particulate or spherical components. We have recently demonstrated a method for computing the refractive indices of spheres composed of polymers measured using mid-infrared point spectroscopy [9]. However, these nonlinear solutions require iterative computation of the forward model. Since Mie theory is computationally expensive, this solution becomes impractical for multidimensional spectroscopic images. In particular, a fast forward model is necessary for iterative calculations of inverse solutions [9], characterization of imaging systems with multiple interacting optical components [10], and iterative fitting of large numbers of particles [11].

### 1.2. Previous Work

Several applications have been developed for computing spectra based on the scattering properties of spheres. Algorithms like MiePlot [12] provide plotting functions for scattering amplitude as a function of wavelength, particle size, and material properties. In addition, many plotting algorithms are available for creating Mie scattering plots for specific cases such as coated [13] and multi-layered spheres in shaped beams [14]. Finite element methods are generally used for non-spherical particles [15], while general solutions are known for fibers [16, 17]. These computational methods are often based on the BHMIE method proposed by Bohren and Huffman [8]. Open-source implementations are also available [18].

However, the available tools focus on creating plots that characterize scattering in highly specific cases, which include incident plane waves or fields focused on the sphere. These cases generally allow for a closed-form analytical solution for the scattered field. We have found no interactive tools for exploring the multidimensional scattered field produced by spheres. This is primarily due to (a) the computational complexity of evaluating a scattered field, (b) the large amount of memory required to store a pre-computed field, and (c) a lack of visualization methods available for exploring simulation results.

Recent methods have been proposed for creating a general framework for simulating scattering in microspectroscopy [16, 19], with the goal of correcting artifacts. These methods evaluate cross-sections of the EM field near the sample. However, these techniques are currently very time-consuming and focus only on the evaluation of scattering through planar substrates.

**FIGURE 1:**
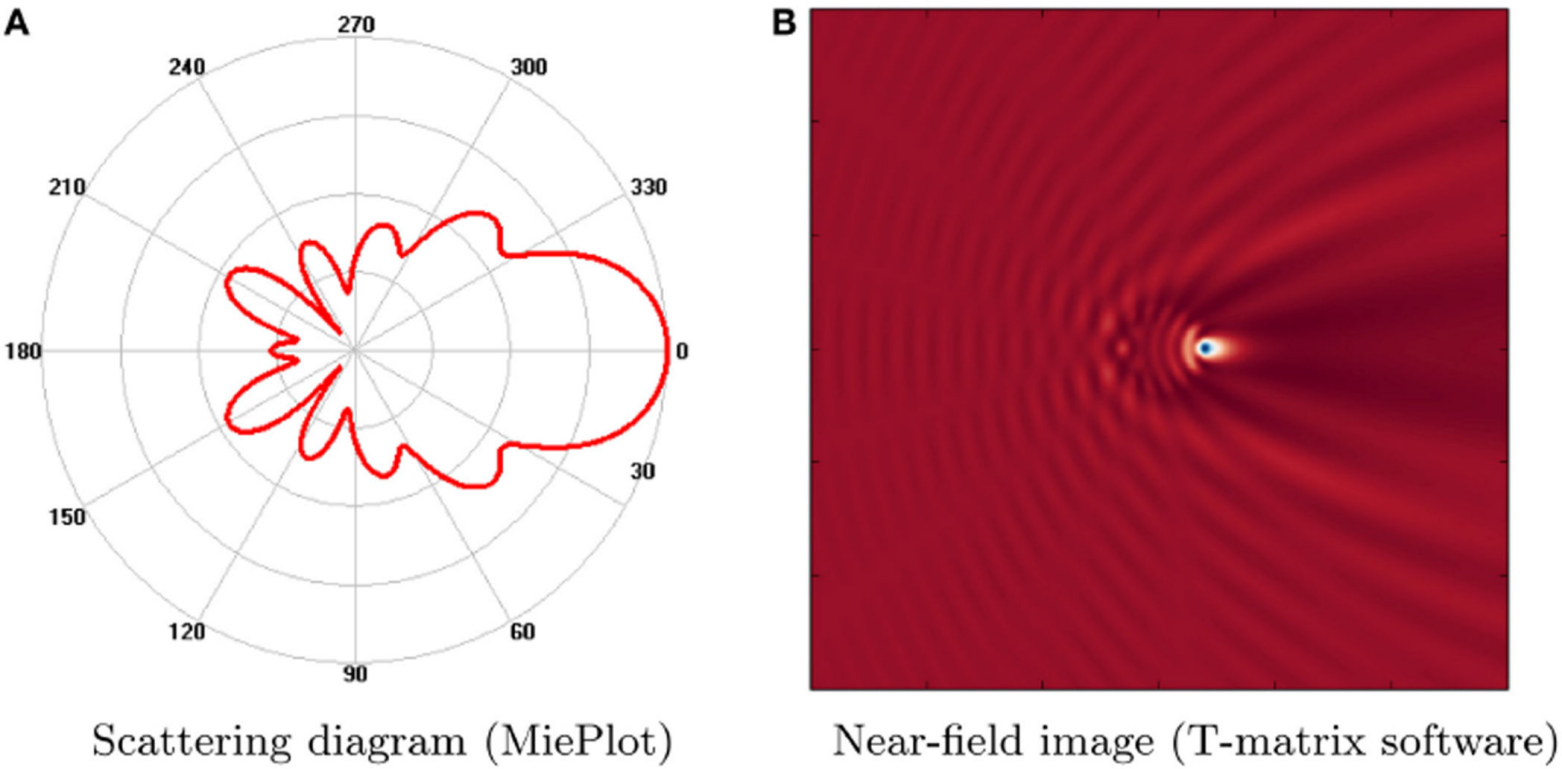
Examples of Mie scattering functionality provided from available tools **(A)** Polar plot of scattered intensity vs. scattering angle (*λ* = 1 µm, sphere radius = 1 µm) created using MiePlot [12]. **(B)** Scattered field produced by a PMMA sphere (radius = 1 µm, *λ* = 1 µm) positioned at the focal point. Image was created using a Python-based GUI for the parallel Multi-Sphere T-Matrix software [20].

Figure 1 shows an example of a polar scattering plot and a near-filed image produced by existing applications. While useful for understanding some Mie scattering properties in specific cases, 2-D scattering plots alone are not sufficient for understanding the multidimensional structure of a scattered EM field. Furthermore, the computational time required by MiePlot [12], for computing scattering plots based on Mie theory (Figure 1A), increases proportionally to the radius of the sphere. The T-matrix software [20] requires 15 s for a full CPU evaluation of scattered fields (600 × 600 slice resolution) at a single wavelength for a sphere positioned at the focal point (Figure 1B).

## 2. MATERIALS AND METHODS

We simulate an optical system similar to those used in most microscopes. The incident light is emitted from a source with an intensity spectrum given by *I*_0_(*k*), where $k=\frac{2\pi }{\lambda }$ is the wavenumber.

Our simulation focuses on the scattering effects of spheres specified by a radius *a* and a complex refractive index *n*(*k*) = *η*(*k*) + *iκ*(*k*). Here, the real part, *η*(*k*), denotes the refractive index and indicates the phase velocity, while the imaginary part, *κ*(*k*), quantifies absorption. The total field resulting from scattering by a single sphere is given by:

(1) $$E(r,\theta )=\{\begin{array}{cc} E_{i}(r,\theta ), & \text{if }r<a\\ E_{f}(r,\theta )+E_{s}(r,\theta ), & \text{otherwise} \end{array}$$

where **E**_*f*_ is the incident field produced by the light source and focusing optics, **E**_*s*_ is the scattered field, **E**_*i*_ is the field inside of the sphere, (*r, θ*) are the spherical coordinates of each point in the field, and *a* is the radius of the sphere. The internal and external fields are equal at the sphere surface: **E**_*i*_(*r, θ*) = **E**_*f*_(*r, θ*) + **E**_*s*_(*r, θ*) for *r* = *a*.

### 2.1. Calculation of the Focused Incident Field (E_*f*_)

The vector formulation for the electric field is given by

(2) $$E_{f}(k,r)=E_{0}e^{ik^{⊤}r}$$

subject to the constraint that **E**_0_^⊤^**k** = 0. Here, **E**_0_ is a vector providing the amplitude and polarization direction of the field, **k** is a vector representing the direction of the incident plane wave, and **r** = (*x, y, z*)^⊤^ is the position vector.

#### 2.1.1. Scalar Model

The focused field is represented using a superposition of plane waves:

(3) $$E_{f}(r)=E_{0}\sum_{j=1}^{J}E_{f}(k_{j},r)=E_{0}\sum_{j=1}^{J}e^{i{k_{j}}^{⊤}r}$$

We implement Debye focusing [21, 22] by first applying the partial wave expansion of the plane wave to obtain

(4) $$e^{ik^{⊤}r}=\sum_{l=0}^{\infty }(2l+1)i^{l}j_{l}(\text{kr})P_{l}(\text{cos }\theta ),$$

where *l* is the order of the incident field, *j_l_*(·) is an order-*l* spherical Bessel function of the first kind, *P_l_*(·) is an order-*l* Legendre polynomial, and *k* = ‖**k**‖ is the wavenumber. We use the *j* subscript to indicate the field produced by a single propagating plane wave **k_j_**. We also assume that the incident light is coherent, therefore ‖**k_j_**‖ = *k* for all *j* ∈ *J*.

**FIGURE 2:**
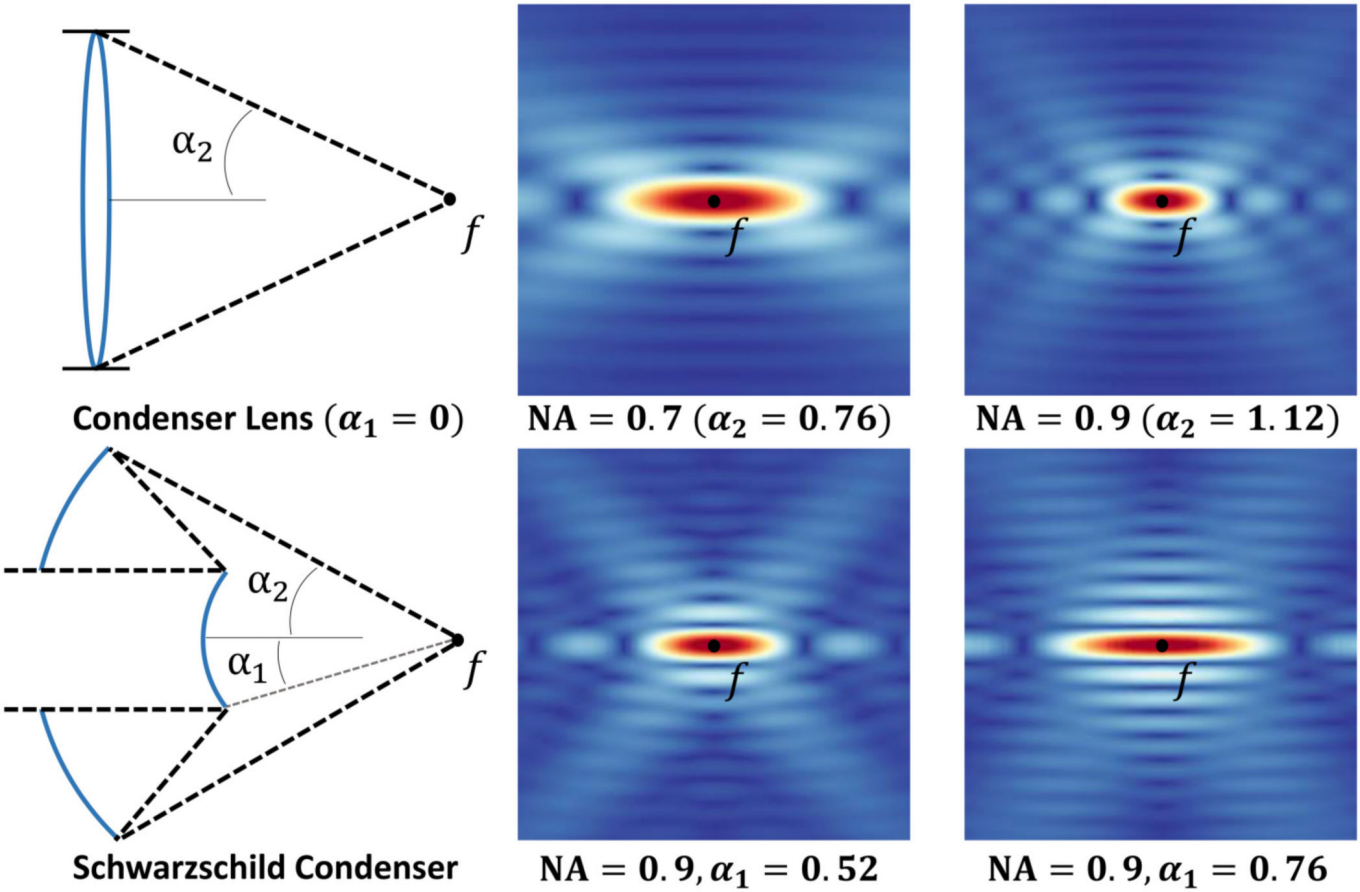
Constructive interference at the focal point *f* results in a high-intensity point spread function (PSF) The magnitude of the field resulting from Equation (6) is shown for a transparent lens **(top)** and a Schwarzschild objective **(bottom)**. Note that increasing the size of the center obscuration creates a PSF with the characteristic features of a Bessel beam.

Using the addition theorem of the spherical harmonics [23] it is possible to write the sum of the plane waves as the following integral

(5) $$E_{f}(p)=2\pi E_{0}\sum_{l=0}^{\infty }(2l+1)i^{l}j_{l}(\text{kr})P_{l}(\text{cos }\theta )\;\int_{\alpha_{1}}^{\alpha_{2}}P_{l}(\text{cos }\theta_{k})\text{ sin}(\theta_{k})d\theta_{k}$$

where *α*_1_ and *α*_2_ indicate the angles subtended by the objective (Figure 2), *θ_k_* is the angle between the unit position vector and the propagation direction of the incident plane wave: $\text{cos }\theta_{k}=\frac{k^{⊤}r}{\left\|k\|\|r\right\|}$, and **p** = (*r, θ*). For a lens, *α*_1_ = 0 and *α*_2_ = sin^−1^(*NA*). In the case of a cassegrain mirror, which is commonly used for mid-infrared spectroscopic measurements, the inner angle *α*_1_ will be the angle subtended by the central obscuration. If *α*_1_ and *α*_2_ are known, the incident field can be computed using a closed-form solution:

(6) $$E_{f}(p)=2\pi E_{0}\sum_{l=0}^{\infty }i^{l}j_{l}(\text{kr})P_{l}(\text{cos }\theta )c_{l},$$

where *c_l_* = [*P*_*l*+1_(cos *α*_1_) − *P*_*l*+1_(cos *α*_2_) − *P*_*l*−1_(cos *α*_1_) + *P*_*l*−1_(cos *α*_2_)]. The solution to Equation (6) for various optical parameters produces the point spread function (PSF) shown in Figure 2.

### 2.2. Calculation of the Scattered and Internal Fields (E_s_ and E_i_)

The scattered field for a single incident plane-wave **k** produced by a sphere with radius *a* positioned at the focal point **p**_*f*_ is given by:

(7) $$E_{s}(p)=\sum_{l=0}^{\infty }B_{l}(\lambda ,n,a)h_{l}^{\left(1\right)}(\text{kr})P_{l}(\text{cos }\theta )$$

where $h_{l}^{\left(1\right)}(x)$ is the order-*l* spherical Hankel function [8]. The coefficients *B* that couple the internal and external scattered fields are given by:

(8) $$B(\lambda ,n,a)=(2l+1)i^{l}\frac{j_{l}(\text{ka})j_{l}^{\prime }(\text{kna})n-j_{l}(\text{kna})j_{l}^{\prime }(k)}{j_{l}(\text{kna})h_{l}^{(1)\prime }(\text{ka})-h_{l}^{\left(1\right)}(\text{ka})j_{l}^{\prime }(\text{kna})n}$$

where $j_{l}^{\prime }(x)$ is the first derivative of the spherical Bessel function of the first kind and $h_{l}^{(1)\prime }(x)$ is the first derivative of the spherical Hankel function. These are derived by enforcing the appropriate boundary conditions [23].

**FIGURE 3:**
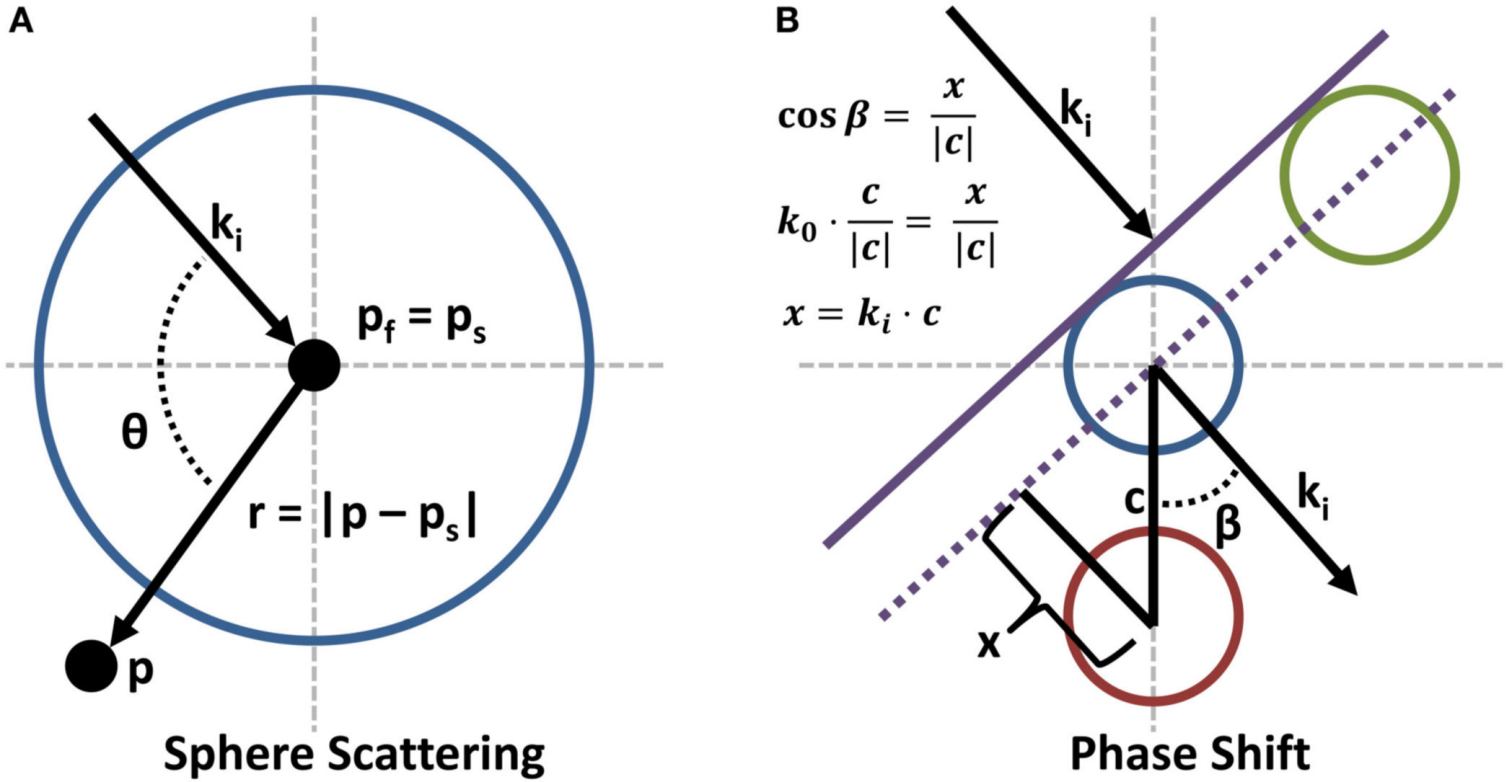
Reference points for scattering through a sphere **(A)** The terms used in the scattering definitions are graphically defined. **(B)** A phase shift is included in the scattering equations for spheres that are not located at the focal point **p_f_**. The purple line gives the advancing wave-front for a plane wave. Note that the green sphere will not require a phase shift, but the red sphere will require a shift equal to *e*^*ik*^⊤^*x*^.

Computing the scattered field for a condenser of *NA_c_* > 0 requires integrating the scattered field equation across the solid angle subtended by the condenser lens. Since the only term in Equation (7) dependent on the light direction is the Legendre polynomial *P_l_*(*cosθ*), this solution can be found analytically. However, this is only valid for spheres centered at **p**_*f*_. For more generally positioned objects, each plane wave must be shifted by an additional phase term in the form of a complex exponential. The need for this phase shift is demonstrated in Figure 3B. A change in sphere position parallel to the direction of propagation of the plane wave will result in a phase delay. Applying this phase shift results in the final scattering equation:

(9) $$E_{s}(p)=e^{ik^{⊤}c}\sum_{l=0}^{\infty }B_{l}(\lambda ,n,a)h_{l}^{\left(1\right)}(\text{kr})P_{l}(\text{cos }\theta ),$$

where **c** = **p**_*s*_ − **p**_*f*_ is the vector from the focal point **p**_*f*_ to the center of the sphere **p**_*s*_.

The internal field, specifying the field inside of a sphere, for an incident plane wave **k** is given by:

(10) $$E_{i}(p)=e^{ik^{⊤}c}\sum_{l=0}^{\infty }A_{l}(\lambda ,n,a)j_{l}(\text{knr})P_{l}(\text{cos }\theta )$$

with the scattering coefficients

(11) $$A(\lambda ,n,a)=(2l+1)i^{l}\frac{j_{l}(\text{ka})h_{l}^{(1)\prime }(\text{ka})-j_{l}^{\prime }(\text{ka})h_{l}^{\left(1\right)}(\text{ka})}{j_{l}(\text{kna})h_{l}^{(1)\prime }(\text{ka})-h_{l}^{\left(1\right)}(\text{ka})j_{l}^{\prime }(\text{kna})n}$$

Note that these equations have no known closed-form integrals. Therefore, the scattered field produced by a focused beam must be computed numerically.

## 3. DISCUSSION

Using the proposed formulation (Equations 6, 9, and 10), we demonstrate interactive performance by minimizing the amount of computation and taking advantage of GPU-based hardware. GPU-based methods provide an inexpensive means of high-performance computing by taking advantage of parallelism and efficient allocation of resources. We are able to achieve interactive performance by minimizing the computational load and limiting the simulation domain to regions visualized by the user. In addition, fast evaluation is useful in situations where iterative evaluation of the forward model is required to solve an inverse problem [9, 11].

### 3.1. Focused Field

Note that the focused field *E_f_* (Equation 6) depends only on position, wavelength *λ*, and the condenser *NA_c_*. We first describe a concise representation of the focused field that allows fast evaluation of *E_f_* as a function of position. We then show that this representation can be constructed quickly, thereby allowing interactive selection of the wavelength and condenser NA.

#### 3.1.1. Representation

We compute and store the field by taking advantage of the symmetry provided using a spherical condenser. In this case, the condenser aperture subtends a circular solid angle that results in a circularly-symmetric incident field centered on the focal point. Note that this formulation is equally valid for a cassegrain with a central obscuration.

**FIGURE 4:**
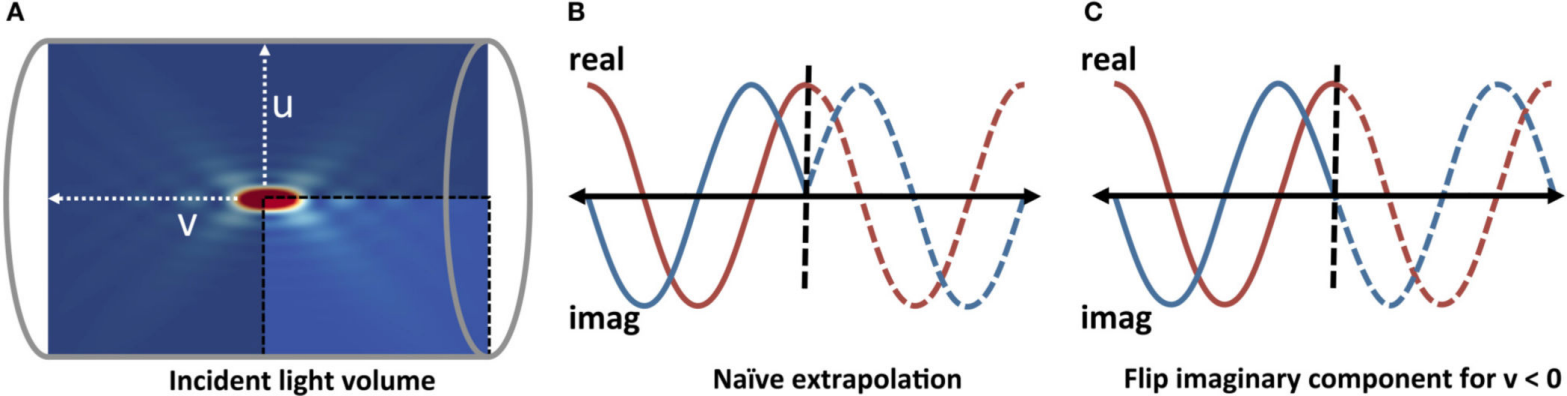
The use of spherical optics to focus the incident light results in a circularly-symmetric incident field E_*f*_ **(A)** A single slice of the incident field is evaluated and stored as a texture map. Any slice through the incident field is computed by interpolating values in this texture using the cylindrical coordinates (*u, v*). **(B,C)** Note that extrapolation across the *v* = 0 plane requires switching the sign of the imaginary component of **E**_*f*_.

Because of this symmetry, the incident field between the condenser and objective can be reconstructed from a single cross-section of the cylindrical volume (Figure 4). This cross-section is stored as a two-dimensional (*R* × *R*) 32-bit floating-point array mapped to a 2-channel GPU-based texture. This texture is referenced in terms of cylindrical coordinates *p* ∈ [*u v*] with the origin located at *p_f_*. In addition, it is only necessary to store $\frac{1}{4}$ of this cross-section by taking advantage of the symmetry along the cylinder axis *v*. Note that direct mirroring of the slice results in a phase shift that is corrected by swapping the sign of the imaginary component for *v* < 0 (Figures 4B,C).

This representation has two major advantages. First of all, hardware-accelerated texture units provide linear interpolation between samples. This reduces the array resolution required to build an accurate approximation of the incident field. Since the simulation domain and resolution are user-specified, the accuracy of the simulation is tightly controlled by defining the desired array resolution *R*.

Secondly, GPU-based texture maps provide spatially coherent caching, which allows a block of processors to acquire values of *E_f_* using a single fetch. Computing *E_f_* at any point in the field then requires at *most* a single texture fetch, and on average $\frac{1}{N}$, where *N* is the GPU warp size (32 on an nVidia GeForce GTX 970). In addition, these texture fetches are performed in parallel to additional computation on newer systems.

#### 3.1.2. Computation

The focused field is evaluated using a GPU-based kernel developed in CUDA. The Legendre polynomials dependent on condenser angle *α* are constant for all points in *E_f_*, and are therefore pre-computed. The second Legendre polynomial, *P_l_*(*cosθ*), depends on position and is computed independently for each point using a recursive definition, requiring only 4 floating point operations for each order *l* =[0 *N_l_*], where *N_l_* is the maximum order of the field (Equation 12).

The final component of *E_f_* is the spherical Bessel function *j_l_*(*k_r_*). These functions are time-consuming to compute. However, the only dependence is distance from the focal point *p_f_*. Since the simulation domain is specified by the user, the parameter-space is one-dimensional and constrained by the user-specified simulation domain and resolution. These values are readily pre-computed and stored in an *N_l_* × *R* table accessed as a 1D 2-channel texture, where *R* is the domain resolution (specified above). This insures at least one sample per pixel.

In conclusion, we show that a high-order representation of the entire field can be pre-computed and stored efficiently when the user makes a change to the condenser NA or incident wavelength. Since the focused field is independent of downstream properties, such as the position and material of particles, the value of *E_f_* is determined at any point using a single texture fetch.

### 3.2. Scattered Field

The implicit functions for the internal field and external scattered field produced by a sphere are given in Equations (9) and (10). The scattered and internal fields are only rotationally symmetric when the position of the sphere is at the focal point (**p**_*s*_ = **p**_*f*_) or when the incident light is represented by a single plane wave $\left(E_{s}=E_{s}^{0}\right)$. This is due to the phase shift introduced by moving a sphere relative to **p**_*f*_ (Figure 3). We utilize the symmetry in the plane-wave solution to represent a particle’s scattering properties in 2D. This image is computed and re-sampled using Monte-Carlo integration to estimate the internal and scattered fields produced by a particle positioned at any point in the near-field.

#### 3.2.1. Evaluation for a Single Plane Wave

The internal and scattered fields are separated into four components:

- the scattering coefficients given by *B* and *A*
- a propagation function given by $h_{l}^{\left(1\right)}$ in **E**_*s*_ (or *j_l_* in **E**_*i*_)
- the Legendre polynomial *P_l_*(cos *θ*)
- the phase shift exponential dependent on the light direction and sphere position.

The scattering coefficients are independent of position and therefore constant for any wavelength *λ* and material *m*. These values are therefore pre-computed for each scatterer. This dramatically improves performance, since each scattering coefficient (Equations 8, 11) requires computing multiple complex-valued Bessel functions and their derivatives.

The attenuation functions $h_{l}^{\left(1\right)}(\text{kr})$ and *j_l_*(*knr*) are dependent on distance from the sphere center. Like the propagation function for the focused field (*j_l_* in Equation 6), both of these parameters are 1D and bounded by *R*. Therefore, these functions are pre-computed for a range of distance values. The Hankel function in *E_s_* is independent of any material properties, and is stored with *j_l_*(*kr*) for the focused field *E_f_* (Section 3.1). Since the Hankel function can be expressed as a linear combination of Bessel functions of the first (*j_l_*(*kr*)) and second kind (*y_l_*(*kr*)), both *j_l_*(*kr*) and *y_l_*(*kr*) are stored in separate channels of the same texture, allowing both attenuation functions for **E**_*f*_ and **E**_*s*_ to be evaluated using a single texture fetch.

The Bessel function used to compute the internal field, however, is dependent on the index of refraction *n*(*k*) of the particle. Therefore, this table is stored for each sphere. The parameter range is significantly smaller, requiring only values *d* < *a*, where *a* is the radius of the sphere. As with the focused field, the Legendre polynomials are computed recursively.

**FIGURE 5:**
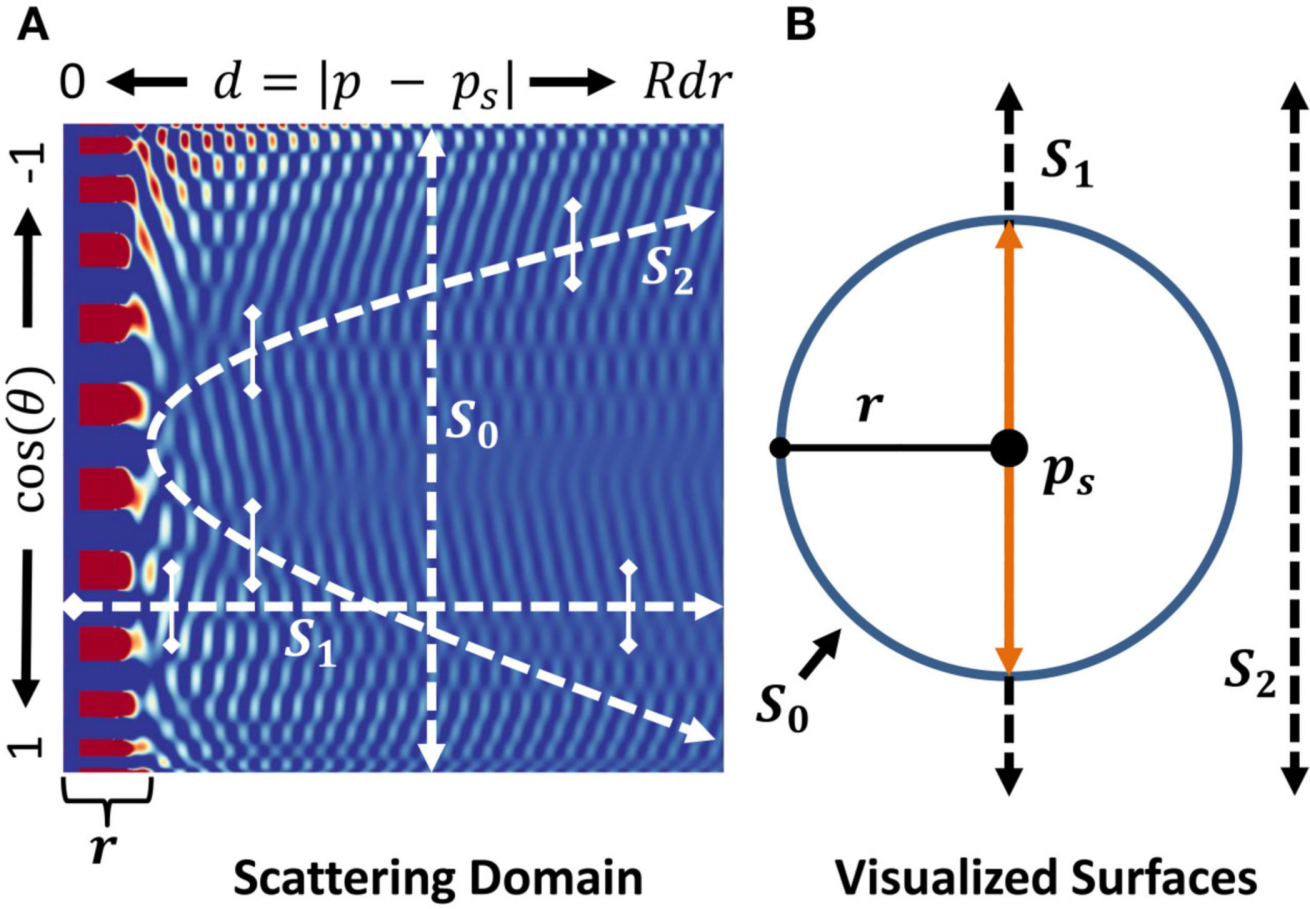
**(A)** The pre-computed scattering domain $E_{s}^{0}$ for a sphere is a 2D function dependent on the position of a point **p** in terms of its distance from the sphere center, *d*, and orientation relative to an incoming plane wave. Color represents field magnitude. **(B)** Surfaces in the near field are shown mapped to the scattering domain. Values where *d* < *r* (orange) use the separate $E_{i}^{0}$ domain. Monte-Carlo samples are always collected along the *y* = *cosθ* direction near the curve, within a range of ±cos *α* as specified by the vertical bars.

These methods are used to compute the *scattering domain* (Figure 5) for a sphere. This a 2D representation of $E_{0}^{s}$ and $E_{i}^{0}$ as a function of $d\in [\begin{array}{cc} 0 & \frac{\text{FOV}}{2} \end{array}]$ and cos(*θ*) ∈ [−1 1]. The accuracy of the simulation is controlled by *R* × *R_θ_*, where *R* is the spatial resolution of the field slice and *R_θ_* is the angular resolution of the scattered field emanating from the sphere surface both inward and outward. The required *R_θ_* is directly dependent on the highest order Legendre polynomial, which has *l* − 1 oscillations in the domain of cos(*θ*). Unlike **E**_*f*_, the internal and scattered fields are scaled by the scattering coefficients *B* and *A*, which quickly converge to zero with increasing values of *l*. The maximum order *N_l_* required for convergence is given by Bohren and Huffman [8]:

(12) $$N_{l}=\lceil \frac{2\pi a}{\lambda }+4\sqrt[{3}]{\frac{2\pi a}{\lambda }}+2\rceil$$

Application of Nyquist sampling implies that *R_θ_* ≥ 2*N_l_* is required to capture all of the oscillations, while greater values increase accuracy for points far from the sphere. Our simulations use *R_θ_* = 1000.

#### 3.2.2. Monte-Carlo Integration

**FIGURE 6:**
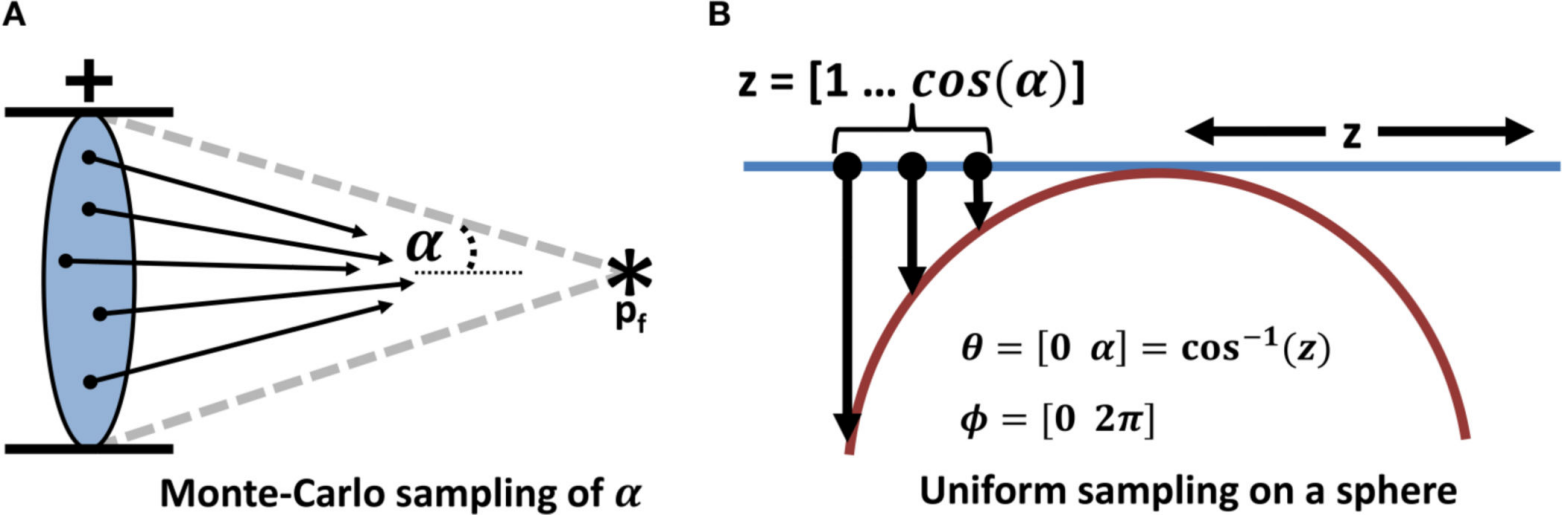
Monte-Carlo sampling of the solid angle *α* defined by the condenser *NA_c_* **(A)** The internal and scattered fields (**E**_*i*_ and **E**_*s*_) are determined by integrating the results based on plane waves originating at points uniformly distributed on the unit sphere and bounded by the condenser aperture. **(B)** Uniform sampling is performed using Archimedes’ principle by creating a uniform distribution on a cylinder in the range of *ϕ* =[0 2*π*], *z* =[1 cos(*α*)] and projecting inward onto the unit sphere.

The solutions for **E**_*i*_ and **E**_*s*_ are determined using Monte-Carlo integration based on a uniform distribution of sample points within the solid angle *α* defined by *NA_c_*. The source points for plane waves are determined using a stratified uniform distribution projected onto the unit sphere based on Archimedes’ principle [24]. A uniform distribution of points is computed in cylindrical coordinates in the range *ϕ* =[0 2*π*], *z* =[cos *α* 1] using jittered stratified sampling [25] and projected inward onto the unit sphere (Figure 6).

The scattered and internal fields produced by a particle are approximated by:

(13) $$E_{s}\approx \frac{2\pi E_{0}((1-\text{cos }\alpha_{2})-(1-\text{cos }\alpha_{1}))}{M}\sum_{j=1}^{M}E_{s}(p,k_{j})$$

$$E_{i}\approx \frac{2\pi E_{0}((1-\text{cos }\alpha_{2})-(1-\text{cos }\alpha_{1}))}{M}\sum_{j=1}^{M}E_{i}(p,k_{j}),$$

where *M* is the number of Monte-Carlo samples, **k_j_** is a single plane-wave direction given by a random sample, and **E**_*s*_, **E**_*i*_ are the equations for a scattered and internal field produced by a single plane wave (Equations 9, 10).

### 3.3. Surface Fields

The scattered field at the sphere surface is an important characteristic, and often the subject of simulations using Mie theory. In addition, both the internal and external fields are identical at this interface. This is generally referred to as the *scattering efficiency*, and is often represented as a graph plot, given as a function of *θ*. However, it is difficult to characterize the scattering efficiency for a sphere when **p**_*s*_ ≠ **p**_*f*_, since this introduces an additional *ϕ*-dependence that is time-consuming to simulate and difficult to visualize using a 1D plot.

**FIGURE 7:**
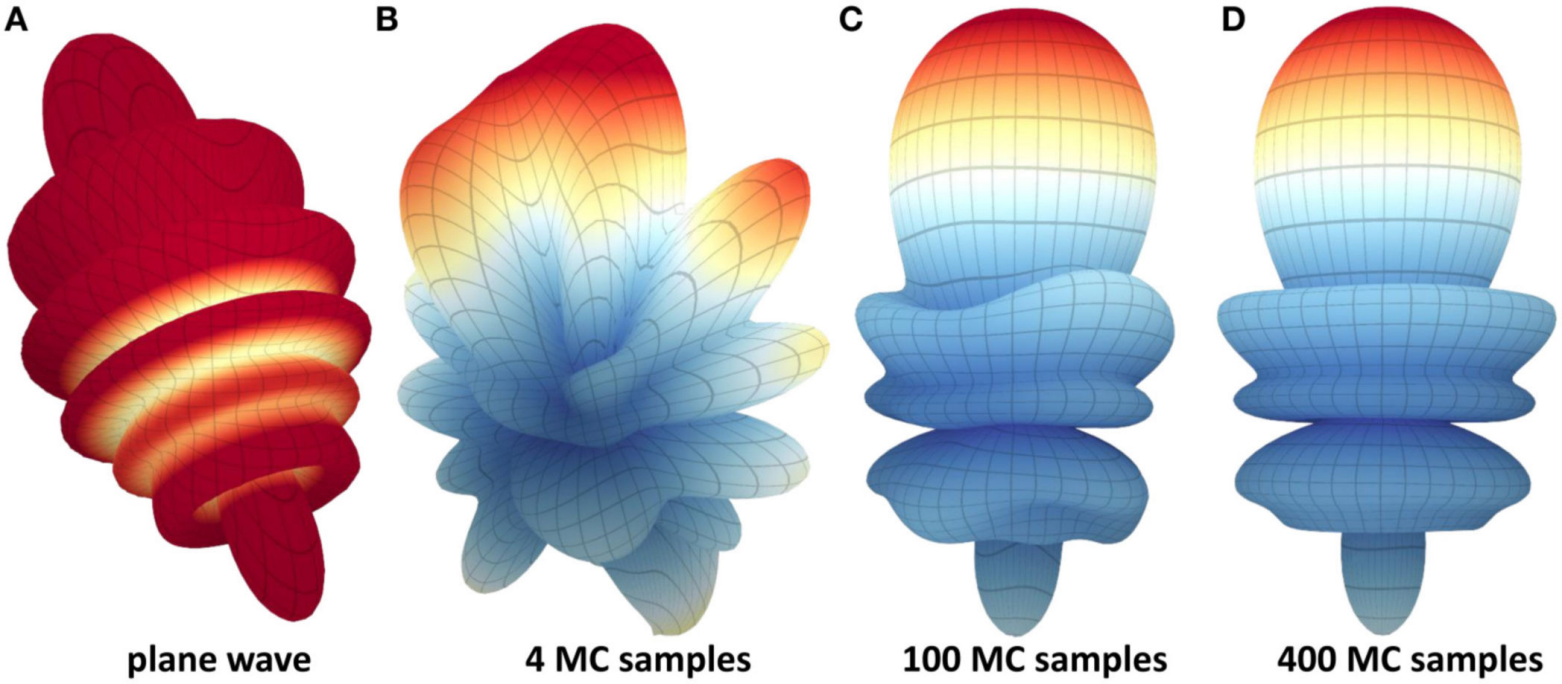
Monte-Carlo sampling of the scattered field at the surface of a 3 µm particle in a *λ* = 2.5 µm field focused by a 0.8 NA condenser The error in Monte-Carlo integration falls off with $\frac{1}{\sqrt{N}}$, where *N* is the number of samples. Due to the constructive coherence in a focused field, the relative error is negligible near the focal point for *N* ≥ 400. The relative error will increase significantly with *d* = |*p* − *p_f_* | since the signal decreases with the square of the distance. **(A)** Scattered field from a simulation using a single plane wave, **(B)** using 4 Monte-Carlo samples, **(C)** using 400 Monte-Carlo samples, **(D)** using 400 Monte-Carlo samples.

We visualize the field using a geometric surface. This technique is similar to those applied to other spherical functions in bio-medical imaging [26], such as diffusion-tensor MRI [27], and provides a unique insight into the structure of the scattered field. This allows the user to explore the scattering efficiency of a particle in three-dimensions as a function of material and position (Figure 9) and validate the effectiveness of MC integration (Figure 7).

### 3.4. Simulated Imaging

The final step in the imaging process is to determine the field at the detector and produce an image. The field is focused onto a detector to produce an image of the objective focal plane. Note that the image plane contains the focal point of the objective lens, *p_o_*.

#### 3.4.1. Objective and Detector

According to the principles of Fourier optics, the objective aperture blocks high- and low-frequency components of the image plane given by the upper and lower cutoff frequencies: $f_{u}=\frac{{\text{NA}}_{o}}{\lambda },f_{l}=\frac{{\text{NA}}_{\text{in}}}{\lambda }$.

The resulting image is produced by evaluating a slice *f*(*x, y*) of the field at *p_o_*. The user specifies the field-of-view (FOV) *S*, and field resolution *R*. We then perform a forward Fourier Transform (FT) of the field slice, resulting in a frequency-domain representation *F*(*u, v*) = ℱ[*f*(*x, y*)]. Frequency components above the cutoff frequency *f_u_* and below the cutoff frequency *f_l_* are eliminated. The field at the detector is therefore *f̂*(*x, y*) = ℱ^−1^ [*F*(*u, v*)*A_p_*(*u, v*)], where the aperture function is given by

(14) $$A_{p}(u,v)=\{\begin{array}{cc} 0, & \text{if }\sqrt{{\left(u\Delta u\right)}^{2}+{\left(v\Delta v\right)}^{2}}>f_{u}\text{ or }<f_{l}\\ 1, & \text{otherwise} \end{array}$$

and $\Delta u=\Delta v=\frac{1}{S}$. The detector then transforms the complex field into the final intensity image *I* = |*f̂*(*x, y*)|^2^.

**FIGURE 8:**
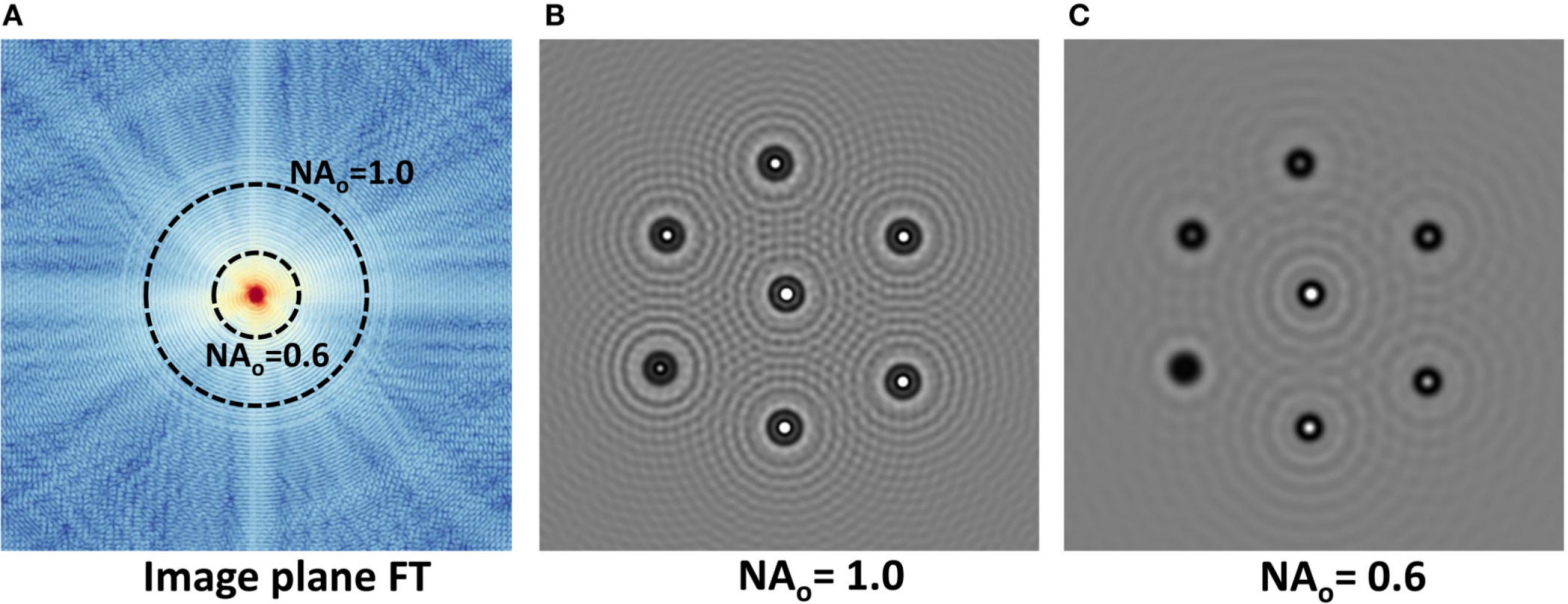
Objective affect on an image of 3 µm particles with *n* = 1.4 + *κ*, where *κ* = 0.0 → 0.12 The incident field is *λ* = 2.5 µm and the field of view is 100 µm. **(A)** Fourier transform of the EM field at the image plane. **(B,C)** Intensity images produced at the detector for 1.0 and 0.6 NA objectives.

The forward and inverse Fourier transforms are performed using the GPU-based cuFFT Fast Fourier Transform (FFT) [28]. The resulting FFT and detector images for an array of particles with varying extinction coefficients *κ* are shown in Figure 8.

#### 3.4.2. Absorbance

Common vibrational spectroscopic imaging techniques, such as mid-infrared spectroscopic imaging, rely on absorbance measurements of a tissue sample in order to estimate the extinction *κ*. The absorption is given by $A=-{\text{log}}_{10}(\frac{I}{I_{0}})$, where *I*_0_ is an image of the incident field, without any particles present.

A simulated image of multiple materials is shown in Figure 10, demonstrating the complex interactions between the incident field, particles, and optics in order to produce a final absorption image.

#### 3.4.3. Extended Sources

We also allow the software to simulate extended (non-coherent) sources. Extended sources are created by simulating multiple point sources and integrating their intensity with the detector. An image of the extended source can be specified, allowing different sources (point, Gaussian, glowbar) to be simulated.

## 4. RESULTS

We have developed a software package called the *Broadband Interactive Mie Simulator* (BIM-Sim), which interactively computes the Bjorn approximation for scattered fields produced by spherical particles. This includes the simulation of a focusing lens, objective lens, and imaging system. The complete field is computed both inside and outside of each particle. BIM-Sim is open-source and available online at http://stim.ee.uh.edu/resources/software/bimsim/.

**FIGURE 9:**
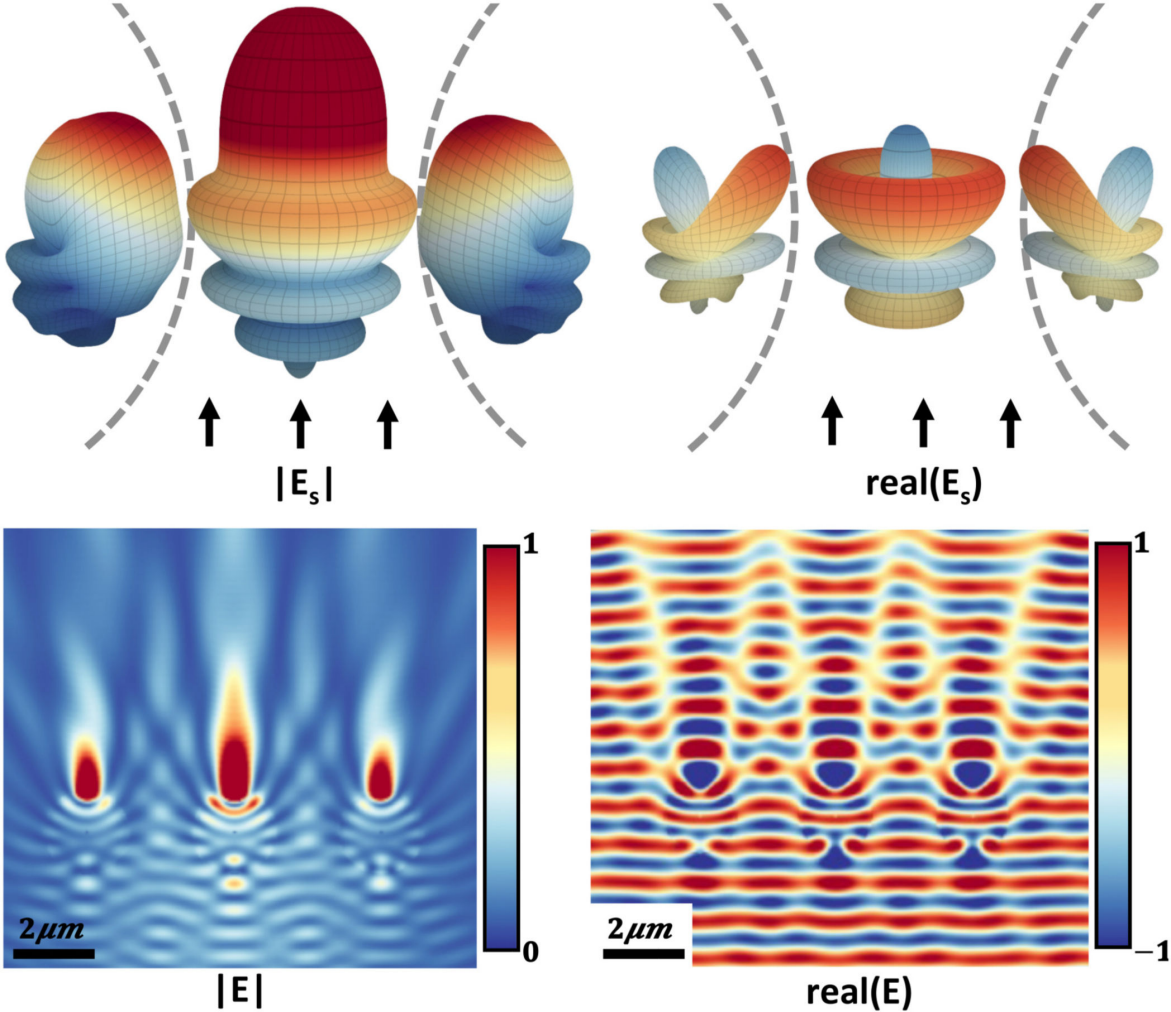
Scattered fields created by three 2 µm diameter spheres separated by 2 µm of vacuum in a focused EM beam produced by a 0.2 NA condenser All three spheres have identical material properties. The magnitude and the real part of the scattered field at the sphere surface are shown **(top)**. Note that the sphere positioned within the center of the focused beam produces radially symmetric scattering, while adjacent spheres show reduced intensity and an asymmetric scattered field. A cross section of the full field (Equation 1) through the spheres is shown **(bottom)**.

We visualize the field using planar cross-sections of the volume placed anywhere between the objective and condenser (Figure 9). The complex components of the scattered field at the particle surface are computed and visualized using a 3D surface model (Figure 9). This facilitates the study of the scattering efficiency of a sphere in three dimensions. The shape of the scattered field is computed using Monte-Carlo sampling, which allows arbitrary positioning of the particle within the focal volume. The use of Monte-Carlo sampling also allows any apodization function to be used for the focusing optics, facilitating the study of beam quality on the resulting field [29, 30].

**FIGURE 10:**
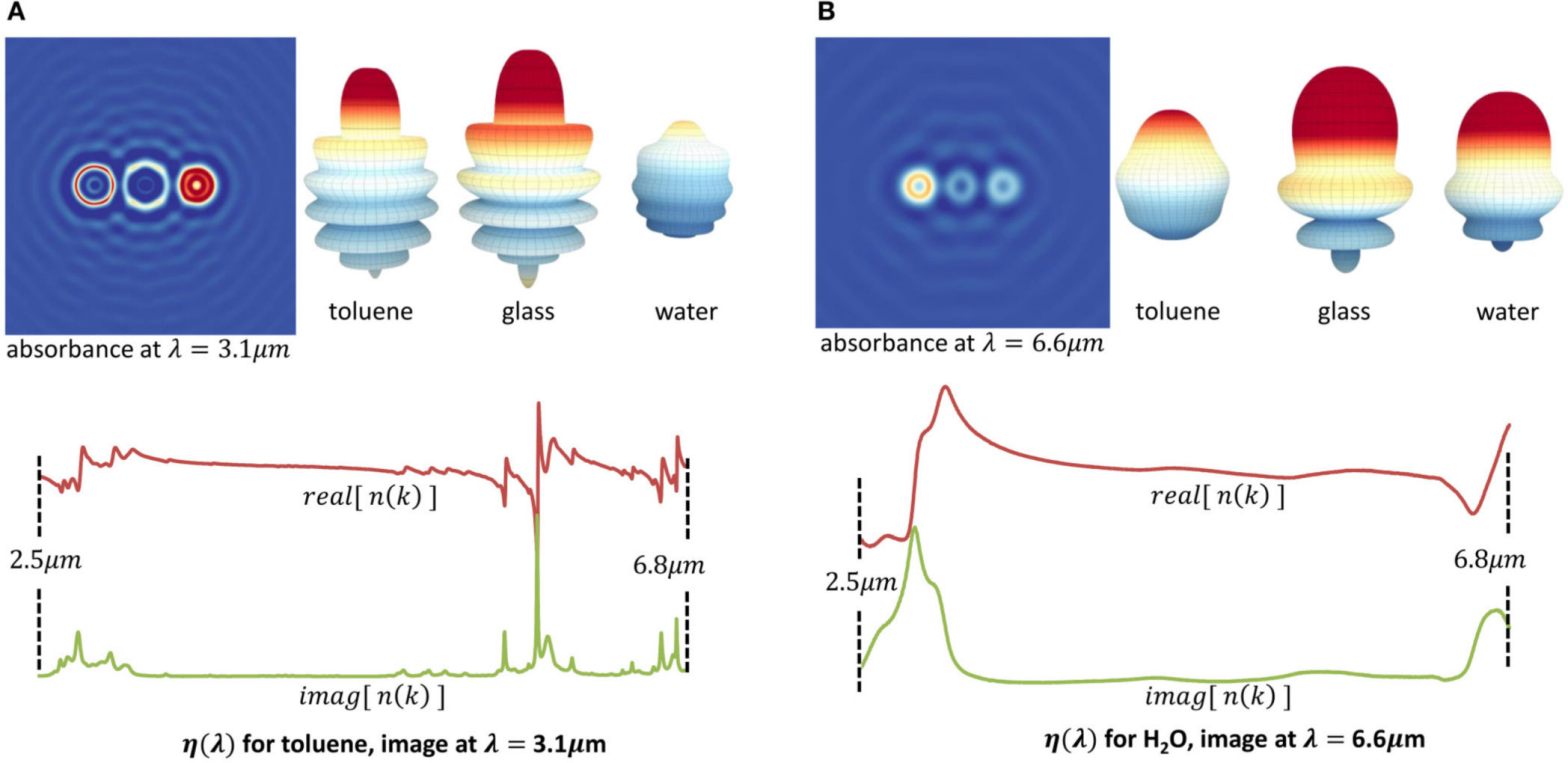
Absorption image and scattering efficiency of three spheres made of different materials with absorption spectra in the mid-infrared **(A)** The absorption image (left) and scattering efficiency (right) is shown at *λ* = 3.1 µm. The real and imaginary parts of the index of refraction are shown for toluene. **(B)** The absorption image and scattering efficiency for *λ* = 6.6 µm is shown (top) with the index of refraction for water (H_2_O, bottom).

**FIGURE 11:**
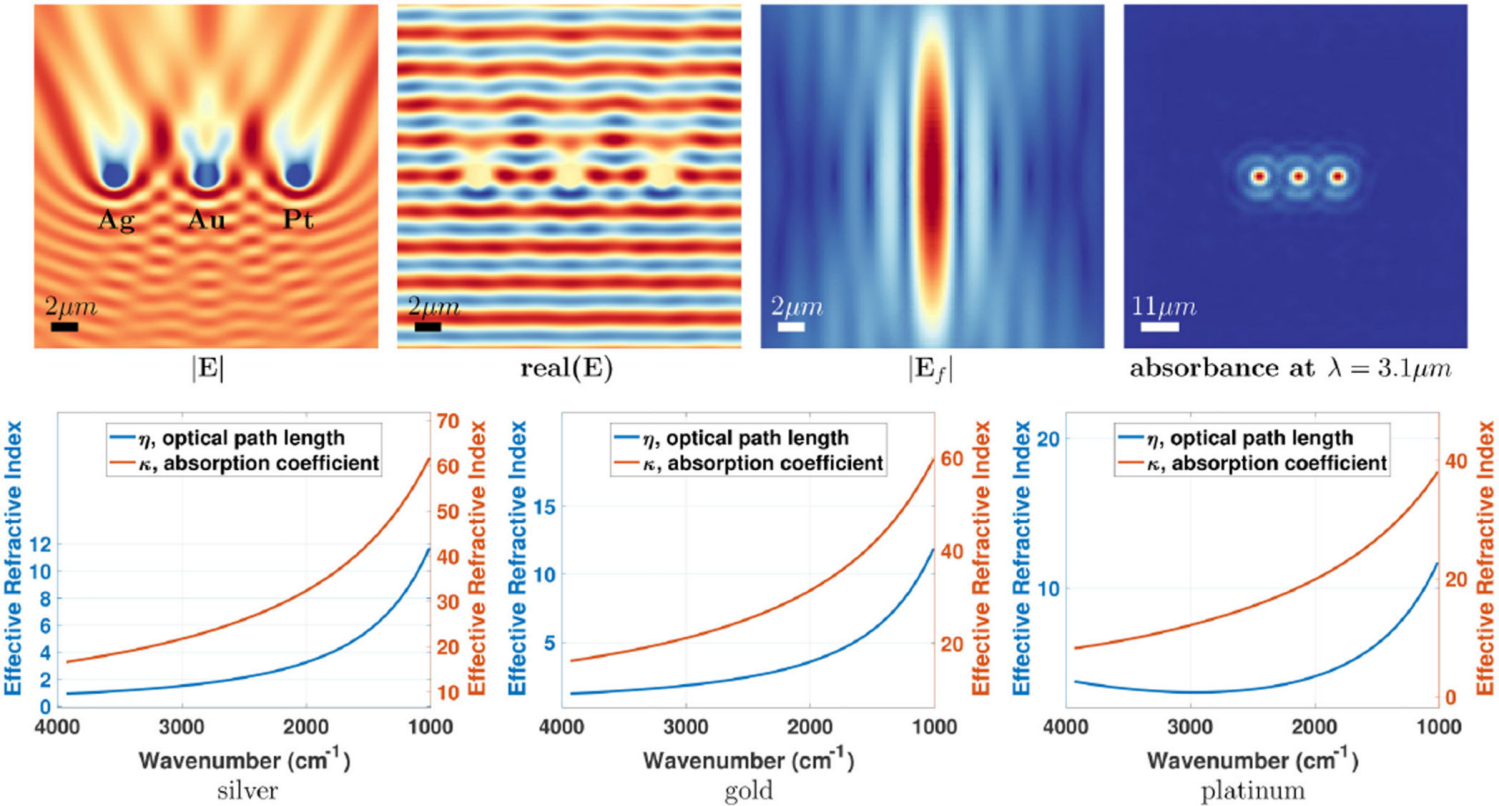
Scattered fields and absorption image created by three 2 µm spheres made of noble metals: silver, gold, and platinum (from left to right) A cross section of the magnitude (|**E**|) and the real part [real(**E**)] of the full field through the spheres, generated by a single plane wave, are shown **(top left)**. The focused EM beam (**E**_*f*_ using a field order of 100) and the absorption image at *λ* = 3.1 µm are shown **(top right)**. The real and imaginary parts of the index of refraction are shown for silver, gold, and platinum **(bottom)**.

The imaging process is simulated by band-limiting a cross-section of the field at the focal plane based on the objective aperture function. The intensity of the filtered cross-section is computed and re-sampled based on user-specified detector parameters to create a final image (Figures 10, 11). Computing both the total and incident field intensity also allows the simulation of absorption spectroscopy for any distribution of micro-spheres. Material properties for the spheres are specified at run-time or as a wavelength-dependent set of refractive indices. If the extinction spectrum is known, BIM-Sim uses the Kramers-Kronig relation to determine the phase speed [31] as a function of *λ*.

### 4.1. Comparisons with Measured Data

We compared the predicted absorbance data (spectra and images) from the forward model described above with IR absorbance data of poly(methyl methacrylate) (PMMA) and polystyrene microspheres. PMMA microspheres were obtained from Bangs Laboratories, Fishers, IN, with diameters between 1 and 6.5 µm. Polystyrene microspheres were obtained from Bangs Laboratories, Fishers, IN, and Polysciences, Warrington, PA, with diameters between 4 and 6 µm. A small volume of spheres was dispersed onto a 3mm thick calcium fluoride (*CaF*_2_) substrate by gently tapping a loaded pipette tip, and regions with individual spheres on the substrate were found. Infrared imaging data were recorded using a Cary 670 Series FTIR Spectrometer coupled to a Cary 620 Series IR Microscope (Agilent Technologies, Santa Clara, CA). The system was equipped with a 128 × 128 pixel focal plane array (FPA) detector that was used for imaging. The NA of the detection Schwarzschild objective and condenser was 0.62 with an obscuration of NA = 0.34. The imaging was performed in high magnification mode (pixel size of 1.1 µm). Spectra were recorded at 16 cm^−1^ resolution.

We estimated the complex refractive index as a function of wavelength from bulk measurements of PMMA and polystyrene absorption spectra. The effective refractive index, *n*, and the absorption coefficient, *k*, obey the Kramer’s-Kronig relations, allowing one to be estimated using a measurement of the other. In this case, these values are computed using the following equations:

$$A(\lambda )\approx -\frac{2\pi }{n_{0}\lambda }\chi \prime \prime (\lambda )n(\lambda )\approx n_{0}+\frac{\chi \prime (\lambda )}{2n_{0}},$$

where *A* is the absorption (per unit thickness), *n*_0_ is the baseline refractive index (≈ 1.4 for most polymers). The real and imaginary components of the magnetic susceptibility

**FIGURE 12:**
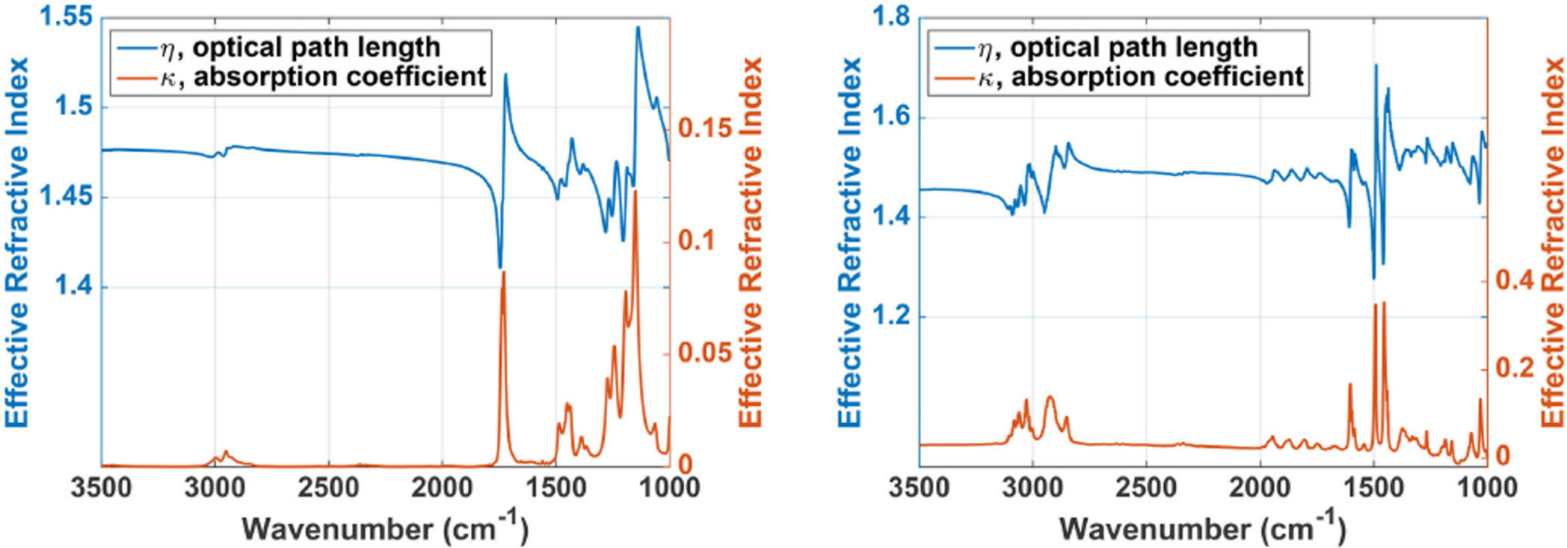
**Complex refractive index for PMMA (left)** and polystyrene **(right)**, computed from absorption measurements collected using FTIR spectroscopy.

χ(λ)=χ′(λ)+iχ″(λ)

are related through the Hilbert transform, and the absorption coefficient

$$k=-\frac{A(\lambda )\lambda }{4\pi }$$

can be computed from the absorption. The complex refractive index for PMMA and polystyrene are shown in Figure 12.

**FIGURE 13:**
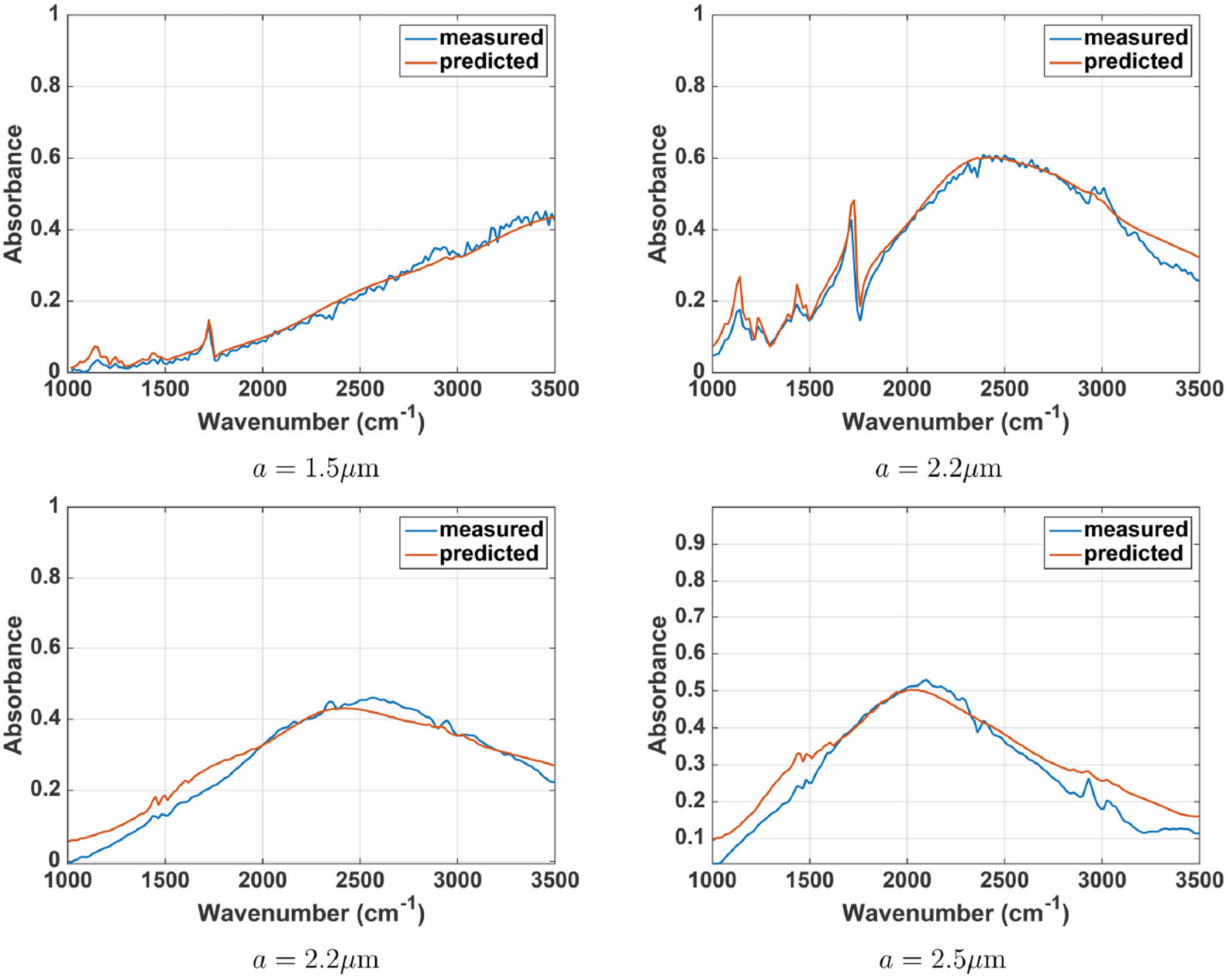
**Measured and predicted absorption spectra for PMMA (top)** and polystyrene **(bottom)** microspheres. Spheres with a radius of 1.5, 2.2, and 2.5 µm are considered. The spectrum from the center pixel of each sphere is shown.

**FIGURE 14:**
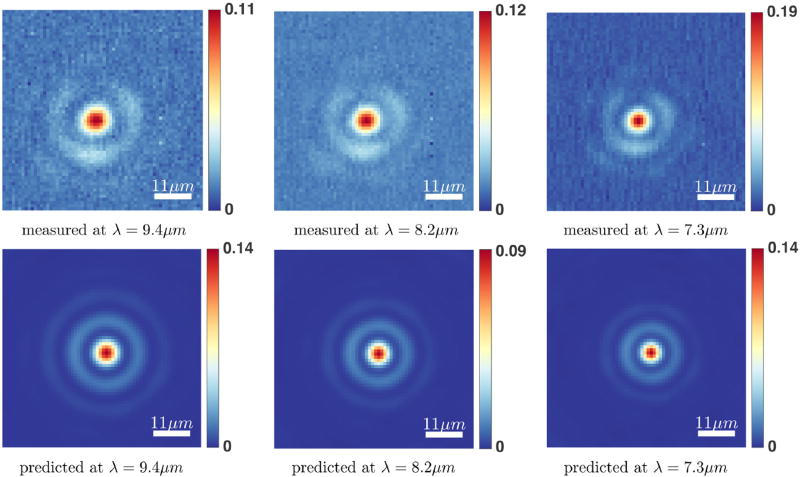
Measured and predicted absorption images for a PMMA microsphere of radius ≈ 2.2 µm at *λ* = 9.4, 8.2, 7.3 µm

**FIGURE 15:**
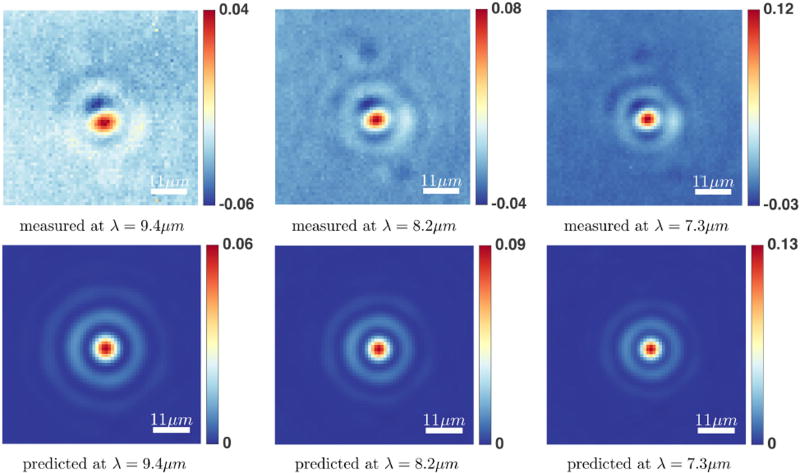
Measured and predicted absorption images for a polystyrene microsphere of radius ≈ 2.2 µm at *λ* = 9.4, 8.2, 7.3 µm

Spectra and absorbance images from a number of PMMA and polystyrene spheres were recorded. The forward model is validated by comparing the predicted absorption spectra of any pixel from the simulated spheres to the actual measured spectra using the FTIR imaging system. The predicted spectra from the described forward model and the measured spectra for PMMA and polystyrene microspheres are shown in Figure 13. The results show a close match between the measured and the predicted spectra. We also compare the simulated absorption images at any wavelength with measured absorbance images (Figures 14, 15). It can be observed that the predicted and measured absorption images match closely up to the noise level.

There are a number of factors which can effect the measured spectrum of polymer microspheres, such as the noise level, position of the sphere in the FOV, the material properties of the sphere, the focal point, the spherical shape of the microsphere etc. We note that the signal-to-noise ratio for the measured data (recorded in high magnification mode) is reasonable but not exceptional while the forward model described here does not incorporate noise. The results shown here are based on running our forward model with the following assumptions about each imaged microsphere: it is positioned at the center of the FOV, it is composed of clear material (in this case PMMA and polystyrene), has a perfect spherical shape, and the microsphere is on focus. We tried to image the microspheres as close as possible to the center of the FOV. However, it was not possible to determine the exact position of the center pixel of each imaged sphere in the FOV using our imaging system (a small offset of the sphere from the center of the image can be observed in Figures 14, 15). The polystyrene microspheres where composed of additional ingredients, such as water and 2.6% latex. The commercially available microspheres are not guaranteed to have a perfect spherical shape and they are of varying diameter size within the same particle solution.

**FIGURE 16:**
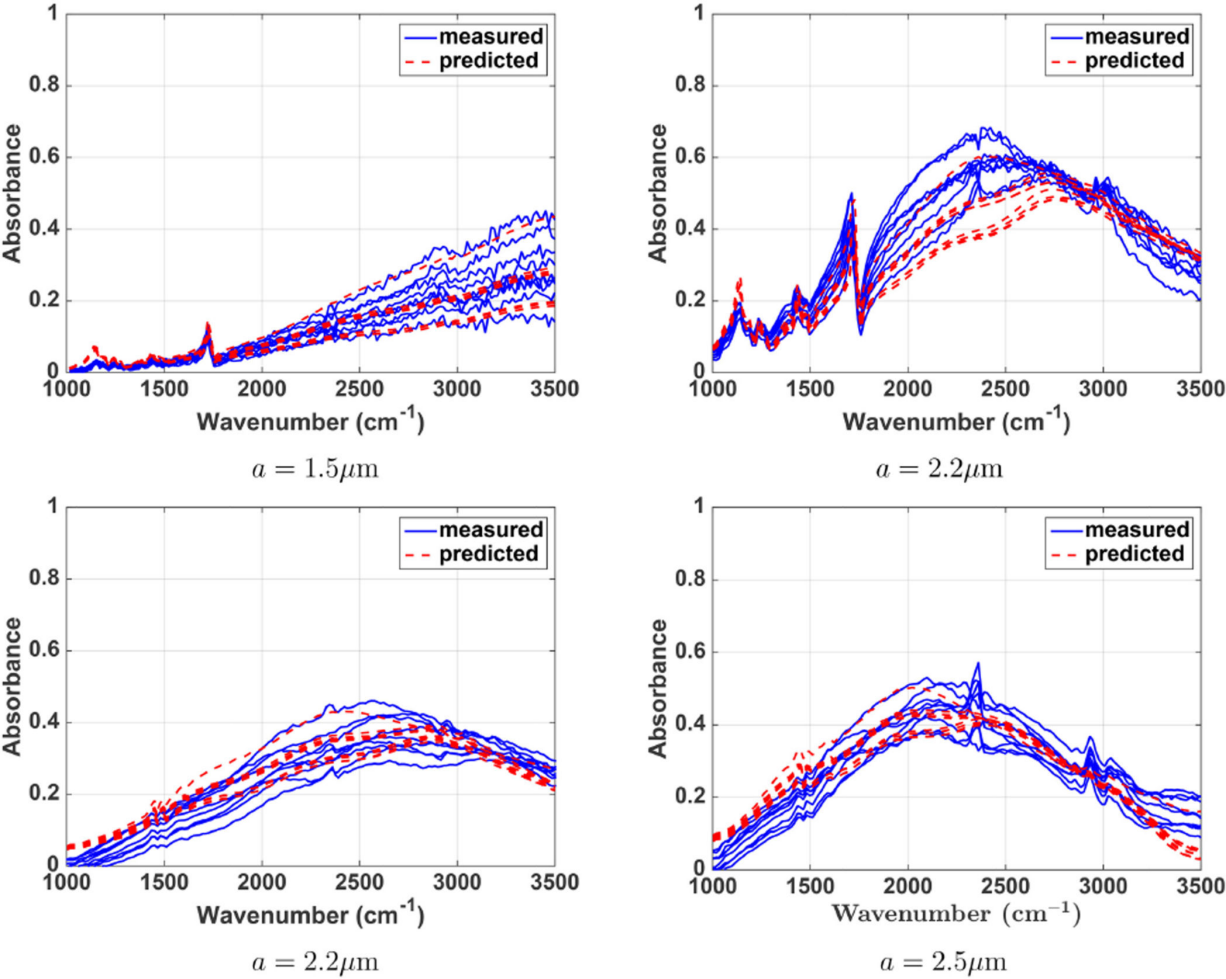
**Measured and predicted absorbance spectra for PMMA (top)** and polystyrene **(bottom)** microspheres. Spheres with a radius of 1.5, 2.2, and 2.5 µm are considered. A cluster of spectra around the center pixel of each sphere is shown.

In order to validate our results for predicted spectra from different pixels in the sphere we compare a cluster of measured and predicted pixel spectra around the center of the spheres. Figure 16 shows that the predicted spectra from different pixel locations closely follow the measured spectra. Also, notice that there is more variation within measured spectra and more overlap within the predicted ones. Overall, our results show that the described theory for the forward model is capable of describing recorded data with commercially available microscopy systems.

### 4.2. Accuracy and Timing Details

The most time-consuming operations are changes to the incident wavelength *λ* and the condenser NA, which require re-evaluation of the focused field and all scattered fields. Our technique allows the EM field to be computed to user-specified accuracy, subject to the limits of numerical precision, since the user can select the resolution of the simulation. We use 32-bit floating point precision.

Stratified sampling is used to limit variance across the condenser aperture [24, 25]. Sample points are reconstructed randomly when *NA_c_* is changed. However, the same random seed value is used, making random samples deterministic in order to reduce flickering as these changes are reflected in the final image.

All of our results were tested on an nVIDIA Geforce GTX 970 with 4 GB of memory and 13 multiprocessors. For a 512 × 512 resolution slice, full evaluation of *E_f_* requires 2–4 ms and evaluation of a single scattered field requires 400–500 ms. This includes both computation of the scatter domain and 400 Monte-Carlo samples of a single sphere. Evaluation time scales linearly with the number of particles. Our current simulations are bounded by the amount of time required to fetch multiple samples of the scattered domain for Monte-Carlo sampling (one fetch per sample).

## 5. CONCLUSIONS

In this paper, we address the need for interactive simulation and visualization of scattered EM fields. This is an important problem in the optics community, where there is significant interest in understanding how micro and nano-particles interact with incident radiation. Our two major contributions include a method for interactive simulation of scattered fields and a framework for visualizing these fields by coupling user interaction with sparse simulation.

The simulation methods that we describe are interactive, allowing exploration of scattered fields that would previously have been impractical. The resulting speedup allows the user to dynamically set all aspects of the simulation. This has many advantages over pre-computation. In particular, a pre-computed representation of a field at the demonstrated magnitude and dimension would require an impractical amount of storage space and would be difficult to query efficiently. In addition, a fast forward model of particle scattering provides a framework for solving inverse problems, such as spectral un-mixing [9] and localization of probes within an imaged field.
